# Personalized nutrition for healthy aging: precision diets based on genomic, metabolic, and microbiome profiles

**DOI:** 10.3389/fnut.2026.1844337

**Published:** 2026-08-05

**Authors:** Claudia Reytor-González, Náthaly Mercedes Román-Galeano, Janeth Castano Jimenez, Oki Yonatan Oentiono, Washington A. Yoong, Martha Montalvan, Daniel Simancas-Racines

**Affiliations:** 1 Center for Evidence Ecosystems, Implementation Science, and Decision-Making (CIDES), Facultad de Ciencias de la Salud y Bienestar Humano, Universidad Tecnologica Indoamerica, Ambato, Ecuador; 2 Facultad de Ciencias Médicas, de la Salud y la Vida, Universidad Internacional del Ecuador UIDE, Quito, Ecuador; 3 Emergency Medicine Department, Indiana University School of Medicine, Indianapolis, IN, United States; 4 Mayapada Kuningan Hospital, Jakarta, Indonesia; 5 Facultad de Medicina Veterinaria y Zootecnia, Universidad Agraria del Ecuador, Guayaquil, Ecuador; 6 Escuela de Medicina, Universidad Espíritu Santo, Samborondón, Ecuador

**Keywords:** aging, gastrointestinal microbiome, genomics, metabolomics, personalized nutrition, precision medicine

## Abstract

The global increase in life expectancy has been accompanied by a parallel rise in the burden of chronic diseases, many of which are influenced by diet and lifestyle. Conventional dietary guidelines, though broadly beneficial, often fail to account for the wide heterogeneity in nutritional needs, metabolic responses, and health trajectories observed among older adults. This limitation has spurred interest in personalized nutrition—a precision approach that leverages individual genomic, metabolomic, and gut microbiome profiles to tailor dietary interventions for healthy aging. Recent advances in omics technologies have deepened our understanding of how genes, circulating metabolites, and microbial ecosystems interact with diet to influence disease risk, physical function, and longevity. This review explores the conceptual foundations, technological enablers, and clinical applications of personalized nutrition, highlighting key evidence from 2020 to 2025. It examines how precision models are being used to improve outcomes in older adults, discusses ethical and social barriers to implementation, and outlines future directions including artificial intelligence and real-time monitoring tools. Personalized nutrition represents a transformative opportunity to shift from population-based dietary advice to individualized strategies that can promote resilience, reduce frailty, and extend healthspan in the aging global population.

## 1 Introduction

According to the World Health Organization, global demographic trends reveal an unprecedented increase in the proportion of older adults worldwide. By 2030, one in six people globally will be aged 60 or above, with the population aged 60 years and older rising from 1.0 billion in 2020 to 1.4 billion in 2030 and doubling to 2.1 billion by 2050. Notably, the number of the “oldest old” aged 80 years and older is expected to triple by 2050, reaching over 400 million. While high-income countries such as Japan already have approximately 30% of their population over 60, the most dramatic growth is occurring in low- and middle-income regions; by mid-century, two-thirds of the world’s older adults will live in these countries (1). This demographic shift is paralleled by a rising burden of chronic noncommunicable diseases (NCDs). Currently, NCDs, primarily cardiovascular disease, cancer, respiratory diseases, and diabetes, account for approximately 75% of global deaths, representing roughly 43 million lives annually. In older age groups, conditions such as type 2 diabetes, heart disease, neurodegenerative disorders, and coexisting morbidities are highly prevalent (2). The convergence of population aging with escalating NCD rates has profound implications for global health systems, emphasizing the urgent need for effective prevention strategies that promote healthy aging.

Diet is a major modifiable factor in the prevention and management of chronic diseases across the lifespan (3). Beyond its role in energy balance and nutrient supply, diet can influence inflammatory pathways, oxidative stress, metabolic regulation, and cellular mechanisms associated with aging. Oxidative stress, defined by an imbalance between reactive oxygen species production and antioxidant defense systems, has been implicated in age-related functional decline and in several chronic conditions linked to aging. In this context, antioxidant-rich dietary patterns, bioactive compounds, and natural products have gained attention for their potential contribution to cellular protection, healthier aging trajectories, and prevention-oriented approaches (4). Recent literature has also highlighted the links between diet, mental health, neuropsychiatric outcomes, and healthy life promotion, suggesting that nutrition-related interventions may be relevant not only for metabolic health but also for cognitive and psychological dimensions of aging (5–7). However, most national and international dietary guidelines are designed as broad, population-level recommendations. While they provide valuable frameworks, for example emphasizing fruits, vegetables, whole grains, lean protein, and limiting added sugars, saturated fat, and sodium, these guidelines often adopt a “one-size-fits-all” approach and may not address the unique characteristics of older adults (8, 9). Noteworthy, older adults are a highly heterogeneous group: chronological age can differ greatly from biological age, and individuals of the same age may vary widely in health status, functional capacity, and nutritional needs (10, 11).

Physiological changes in later life also alter nutrient requirements. Aging is associated with reduced basal metabolic rate due to loss of lean muscle mass, changes in body composition, impaired absorption of some vitamins and minerals, alterations in taste and appetite, and greater prevalence of chronic conditions that influence metabolism, such as insulin resistance and digestive disorders (12). Additionally, older adults may have differential dietary priorities, for example, preventing muscle wasting and frailty requires sufficient protein and micronutrients even at lower energy intake (13). The risk of both undernutrition, including malnutrition and vitamin deficiencies, and overnutrition, including obesity combined with sarcopenia, can coexist in this age group. Yet generalized guidelines rarely account for these complexities (9, 14, 15).

Moreover, many dietary recommendations have been developed with a focus on the average adult, potentially overlooking subgroups. For example, the Recommended Dietary Allowance (RDA) for protein is typically set at 0.8 g/kg body weight, but many geriatric nutritionists argue that older adults may need 1.0–1.2 g/kg per day, or higher intakes under specific clinical or functional conditions, to preserve muscle mass and function (16, 17). Social and cultural factors, including food preferences and meal patterns, as well as health status, comorbidities, and medication use, further complicate adherence to standard recommendations (18). Taken together, these limitations mean that generalized dietary guidance may be insufficient to optimize health in older individuals. A growing body of literature supports the need for more targeted, individualized nutritional strategies to meet the specific challenges of aging (19, 20).

In response to the limitations of broad guidelines, the field of precision nutrition is gaining momentum as a transformative paradigm. Precision nutrition (also called personalized nutrition) integrates genetic, metabolic, microbiome, and lifestyle information to tailor dietary advice to the individual (21). For instance, one individual’s blood sugar or lipid response to the same meal can differ markedly from another’s due to differences in gut microbiota composition, genes governing metabolism, current health status, and other factors (22, 23). In practical terms, precision approaches might use genomic data such as single-nucleotide polymorphisms affecting nutrient metabolism, metabolomic profiles, or even continuous glucose monitors to fine-tune dietary targets for each person. The goal is to enhance diet effectiveness and adherence by aligning recommendations with one’s unique biology and lifestyle (24).

Emerging evidence underscores the potential of precision nutrition to improve health outcomes. For example, gut microbiome-based dietary interventions have been proposed to prevent age-related metabolic diseases. One recent review identified a gut microbiota profile associated with type 2 diabetes prevention in older adults, suggesting that dietary modulation of the microbiome could help ward off diabetes while promoting healthy aging (25). Such strategies recognize that prebiotics, probiotics, or specific nutrients can preferentially promote beneficial bacteria or metabolic pathways in an individual’s gut, thereby improving glucose metabolism and longevity. In addition, dietary patterns and bioactive nutrients, including omega-3 fatty acids and plant-derived compounds, may contribute to anti-inflammatory and antioxidant pathways relevant to cardiometabolic and cognitive health (4, 26). In short, precision nutrition allows personalization of dietary recommendations: not only what nutrients are needed, but in what form or delivery to optimize benefit, and which factors, including dietary habits, microbiota, genetics, and metabolic status, should guide choices (27–29).

Crucially, precision nutrition is supported by rapid technological advances. High-throughput genomics and metabolomics now enable detailed phenotyping of individuals, while wearable sensors and mobile health apps can track diet, activity, and physiological responses in real time (30, 31). Integrating this multi-omics and lifestyle data with artificial intelligence (AI) and machine learning (ML) has shown promise for predicting individual responses. For instance, machine-learning models have been used to predict postprandial glycemic responses based on a person’s health parameters and gut microbiome, outperforming average predictions (32, 33). These computational tools help translate vast individual-specific information into actionable diet plans. Early pilot studies using such algorithms have reported modest improvements in glucose control and metabolic health compared to standard advice, though more research is needed (34).

Global experts are actively exploring how precision nutrition can be applied to improve aging outcomes. Workshops and reviews emphasize that personalized dietary strategies could maintain muscle mass, cognitive function, and functional independence in seniors (35–37). Incorporating microbiome analysis may pinpoint optimal fiber sources or fermented foods for each individual to reduce inflammation (38). Although precision nutrition research is still evolving, early studies in diverse populations (including long-lived Asian and European cohorts) suggest substantial inter-person variation in dietary needs (39), reinforcing that a targeted approach could better promote health span, the years lived in good health, rather than merely extending lifespan, a distinction consistent with the Global Burden Disease framework and its emphasis on disability-related burden through years lived with disability and disability-adjusted life years (40).

This review explores the conceptual foundations, technological enablers, and clinical applications of personalized nutrition, highlighting key evidence from 2020 to 2025. It examines how precision models are being used to improve outcomes in older adults, discusses ethical and social barriers to implementation, and outlines future directions including AI and real-time monitoring tools.

## 2 Conceptual framework: what is personalized nutrition?

Personalized (or precision) nutrition refers to the practice of tailoring dietary advice and interventions to accommodate an individual’s distinct nutritional requirements, genetic composition, health condition, lifestyle, and personal preferences (41). This inherently acknowledges inter-individual variability in nutrition needs, which may be influenced by age, sex, body composition, metabolic rate, genetics and other factors, and aims to deliver tailored and effective nutritional recommendations that promote health outcomes (42, 42). Thus, the core principle of personalized nutrition is that individuals with distinct genetic and biological characteristics may respond differently to dietary interventions, and that leveraging individual-specific information can enhance the relevance and effectiveness of nutritional guidance (43).

At the center of this framework is the notion that population-wide dietary guidelines can be translated into individually optimized diets (44). By integrating a person’s genetic polymorphisms, biomarkers, and lifestyle data, personalized nutrition builds upon established population-level dietary guidelines while tailoring recommendations based on an individual’s specific characteristics. In effect, it aims to transform traditional food guidance into genome-guided nutrition strategies for each individual (41). For example, nutrients that are beneficial on average might be adjusted or replaced depending on one’s genetic predisposition or metabolic profile. Emerging thought leaders in the field describe personalized nutrition as a discipline that leverages genetic, phenotypic, biochemical and even sociocultural information to tailor diet, anticipating that this will improve healthspan and potentially reduce healthcare costs in the long run (27). In this way, the conceptual framework of personalized nutrition is built on the principle of individualization: acknowledging diversity in response to diet and applying advanced data to create more precise nutritional plans (45).

### 2.1 Enabling technologies

Advancing personalized nutrition depends on technologies that can measure, integrate, and interpret individual biological variability (45). A holistic multi-omics approach has been proposed as a conceptual framework that integrates of genomics, transcriptomics, proteomics, metabolomics, metagenomics (microbiome) and epigenomics (46, 47). Combining these complementary layers may improve the identification of pathways underlying nutrient metabolism, disease susceptibility, and responses to dietary interventions.

In this context, next-generation sequencing enables the incorporation of the gut microbiome into multi-omics models. Whereas 16S ribosomal RNA gene sequencing primarily characterizes microbial composition, shotgun metagenomics provides greater taxonomic resolution and enables the assessment of microbial genes and functional pathways. This is particularly relevant to personalized nutrition because metagenomic analyses can identify functions involved in fiber degradation, short-chain fatty acids (SCFAs) production, bile-acid metabolism, and other diet-related processes. Large-scale microbiome initiatives, including MetaHIT and the Human Microbiome Project, have generated microbial gene catalogs and reference datasets that support functional interpretation. When combined with clinical, dietary, genomic, and metabolic data, these resources may help clarify individual differences in nutrient utilization (48). More broadly, personalized nutrition frameworks integrate multiple data layers to characterize an individual’s nutritional phenotype. In essence, no single data type can fully explain health or dietary responses, combining genomic, metabolomic, proteomic, microbial, and related data may provide a more comprehensive basis for linking individual biology with tailored dietary strategies (49).

### 2.2 Genomics and nutrigenomics

Genetic analysis forms a foundational layer of personalized nutrition. Nutrigenomics/nutrigenetics investigates how genetic variants influence dietary responses (50, 51). For example, single-nucleotide polymorphisms can determine how one metabolizes fats, carbohydrates, vitamins or other nutrients. Personalized nutrition uses this information to refine diet plans (44). Through the assessment of an individual’s genetic profile, dietitians can modify macronutrient ratios or supplement needs according to inherited factors. In principle, this could improve outcomes by aligning diets to genetic risk: as one review notes, nutrigenetics is the science of linking gene variants to differential nutrient responses, and personalized nutrition recommendations based on the knowledge of an individual’s genetic background may enhance the effectiveness of specific dietary interventions (52). In practice, some interventions incorporating genetic data have shown promise. For instance, an exploratory European study found that older adults who received personalized advice based on genetic and physiological information reduced their body fat percentage more than those receiving standard advice (53). Though challenges remain (nutritional phenotypes often involve many genes of modest effect), genomic data provide an opportunity to tailor dietary recommendations beyond generalized nutritional guidelines. Thus, nutrigenomics enables personalized nutrition to move from population averages toward individualized precision, accounting for inherited differences in nutrient metabolism and disease risk (54, 55).

### 2.3 Metabolomics

Metabolomic profiling constitutes a key element in the implementation of personalized nutrition strategies. Metabolomics measures the small-molecule metabolites (e.g., amino acids, lipids, vitamins, and others) present in an individual’s blood, urine or tissues (56). These metabolites serve as indicators of the interactions between dietary intake and biological processes. As one review points out, metabolomics provides biochemical insights in conjunction with genomic and proteomic data, thereby establishing a direct correlation with the phenotype of an organism (41). In other words, metabolomic analysis identifies how a person’s body responds on a molecular level to foods and nutrients. The objective is to translate those response patterns into targeted dietary modifications. For example, metabolite signatures can reveal hidden nutrient deficiencies or metabolic blockages, allowing for targeted changes (such as increasing particular amino acids or altering fat intake) (57). Taken together, metabolomics complements genetics by capturing dynamic biochemical responses to dietary intakes, thereby enabling the refinement of nutritional recommendations based on observed metabolic outcomes rather than solely on genomic predictions.

### 2.4 Microbiome profiling

The gut microbiome, defined as the community of microbes living in the digestive tract, is a key biological factor in personalized nutrition (25, 58, 59). The composition of the gut microbiota exhibits substantial interindividual variability and plays a significant role in modulating responses to dietary interventions. Gut microbes contribute to digestion, nutrient absorption and production of bioactive compounds (such as vitamins, SCFAs, and other metabolites) (60). Importantly, a bidirectional relationship exists between diet and the gut microbiota, whereby dietary intake influences the composition and function of the microbiome, while the microbiota, in turn, modulates the host’s response to dietary components (61). As noted in recent literature, the microbiome can impact host physiology through its role in digestion and nutrient bioavailability. Personalized nutrition leverages this interplay by profiling a person’s microbiome and then prescribing dietary modifications to optimize it (62). For example, if sequencing shows a low abundance of fiber-fermenting bacteria, a practitioner might recommend specific prebiotics or high-fiber foods to promote those microbes (63, 64). According to recent literature, gut microbes affect host nutritional status by influencing energy extraction and nutrient bioavailability, so microbiota modulation could contribute to meet nutritional requirements and help address malnutrition or metabolic disease (37). In practice, interventions like tailored probiotic or fiber regimens are being studied to shift individual gut ecology in beneficial ways. Consequently, microbiome profiling provides valuable insights for personalized nutrition by elucidating individual host–microbe interactions, thus enabling the development of tailored dietary strategies aimed at promoting a healthier microbial composition and improving digestive and metabolic health. In this context, population-level microbiome profiles can provide reference patterns of microbial diversity and functional capacity, helping to interpret whether an individual microbiome deviates from expected healthy or age-related configurations; however, their use in personalized nutrition remains interpretative rather than prescriptive and should be integrated with dietary, metabolic, clinical, and lifestyle data (48).

### 2.5 Digital tools and AI

Beyond laboratory measurements, modern personalized nutrition also draws on digital health and computational technologies. Mobile and wearable devices now allow continuous monitoring of diet and lifestyle inputs, and advanced software can process that data to give customized feedback (65, 66). In the digital era, users increasingly rely on smartphone apps and sensors for health advice (67). These applications monitor multiple aspects of daily life, including physical activity and dietary intake; collect user-specific data, and employ advanced data-driven approaches, such as AI and ML, to generate personalized dietary and lifestyle recommendations (67, 68). For example, a nutrition app might analyze a week’s food logs and use ML to predict how certain meal changes would affect that user, then suggest personalized meal plans (68). Advanced concepts like the “personal digital twin” have been proposed, where an AI model of an individual’s metabolism is continuously updated with sensor data, allowing real-time refinement of diet recommendations (69). In this way, informatics and AI extend personalized nutrition by automating the integration of complex data streams and delivering adaptive, individualized guidance.

### 2.6 Benefits compared to traditional dietary guidelines

Personalized nutrition is envisioned to offer distinct advantages over conventional, population-based dietary guidelines. By accounting for an individual’s unique biological and lifestyle context, personalized nutrition can target personal risk factors and nutrient needs that generic advice cannot address (43). By identifying such factors, a personalized approach can adjust macronutrient ratios or micronutrient intakes appropriately. The Food4Me randomized controlled trial found that individuals who benefited most from an internet-based personalized nutrition intervention were typically older adults, women, those with poorer diet quality at baseline, and those with higher self-efficacy and motivation to eat healthily (70). Notably, the observed benefit appeared to be driven more by baseline behavioral and psychosocial characteristics than by genotype or most demographic factors. Clinically, these findings suggest that personalized nutrition interventions may be particularly effective when directed toward individuals with poorer initial diet quality but strong intrinsic motivation for health improvement. Incorporating these psychosocial and behavioral profiles into future public health and clinical nutrition programs may enhance intervention impact and cost-effectiveness (71).

Despite these potential benefits, evidence from trials is still emerging. A systematic review reported that personalized nutrition interventions have not consistently shown greater improvements in most health outcomes compared with general dietary advice (72). Owing to the complex and multifactorial nature of nutrition, health outcomes are influenced by a wide range of biological, behavioral, and environmental factors. Consequently, personalized nutrition is best regarded as a complementary strategy that refines and extends existing dietary guidelines rather than serving as a wholesale replacement for them. In practice, clinicians might continue to use population-based recommendations as a baseline, then refine them using individual data (44, 72). While generic guidelines remain essential for broad public health messaging, personalized nutrition strategies can fill in the gaps for those with special needs or strong personal goals.

## 3 Genomic profiles and nutritional needs

### 3.1 Genomic profiles and personalized nutrition in older adults

Nutritional genomics examines how genetic variation shapes nutrient metabolism, requirements, and health outcomes. This field encompasses nutrigenetics (how gene variants affect nutrient handling) and nutrigenomics (how nutrients influence gene expression) (50). In practice, personalized nutrition assumes that identical diets can yield different metabolic effects in different individuals due to genetic diversity (21). For example, older adults frequently exhibit high variability in nutrient absorption, metabolic rate, and disease susceptibility (73); thus genomic information may allow tailoring of dietary recommendations to promote healthspan. For example, recent evidence suggests that genetic differences may influence individual responses to dietary interventions, including changes in body weight and body fat percentage, compared with standard dietary advice (74).

One paradigmatic gene–nutrient interaction involves the methylenetetrahydrofolate reductase (*MTHFR*) gene and folate metabolism (75, 76). The *MTHFR* gene encodes methylenetetrahydrofolate reductase, a critical catalyst in the conversion of folic acid to 5-methyltetrahydrofolate (77). The common C677T polymorphism substantially reduces this enzyme’s activity. Individuals homozygous for the 677 T allele typically have 5–30% lower serum folate and 5–6% reduced vitamin B₁₂, along with markedly elevated homocysteine levels (78). Elevated homocysteine is a well-established risk factor for atherosclerosis, stroke, and other age-related vascular diseases (79). In older adults, who are already prone to suboptimal B-vitamin status, *MTHFR* risk genotypes can exacerbate homocysteine accumulation (80). Nutrition-based mitigation strategies have been studied in this context: carriers of the *MTHFR* 677 T allele often show greater need for folate or supplemental 5-methylfolate to normalize homocysteine (81). Indeed, trials have demonstrated that TT homozygotes are less responsive to ordinary folic acid doses, suggesting a requirement for higher or more bioavailable folate (81). Thus, genotyping *MTHFR* can identify older individuals who may benefit from tailored B-vitamin intake (particularly folate and B₁₂) to maintain methylation pathways and limit vascular risk (82).

The fat mass and obesity-associated (*FTO*) gene provides another example of genotype-driven nutrient management. Certain *FTO* variants (e.g., the A allele of rs9939609) are linked to dysregulated appetite and obesity; carriers tend toward higher caloric intake and fat consumption (83–85). Importantly, adherence to a high-quality diet may mitigate the effects of genetic predisposition. In a seven-country adult cross sectional study (the Food4Me study), participants whose diets were low in energy-dense “discretionary” foods (especially those high in saturated fat) and higher in fiber maintained healthier weight irrespective of *FTO* status (86). That is, even *FTO* risk homozygotes benefited from a nutrient-rich, fiber-dense eating pattern. This suggests that standard dietary strategies (such as reducing saturated fat and sugar and increasing whole grains and vegetables) remain effective in genetically susceptible older individuals. In practical terms, knowing a patient’s *FTO* genotype can reinforce the importance of portion control and high-fiber diets for weight management. For example, older adults carrying *FTO* risk alleles might be counseled to monitor portions of refined grains and fatty snacks more carefully, and to prefer high-fiber foods, to counteract this genetic predisposition (87, 88).

Apolipoprotein E (*APOE*) genotype is a third critical factor in personalized nutrition, especially given its influence on lipid metabolism. There are three common *APOE* alleles (ε2, ε3, ε4) that differentially affect cholesterol and lipoprotein handling (89). The ε4 allele, in particular, is associated with higher plasma total and low-density lipoprotein cholesterol (LDL-C) and lower high-density lipoprotein cholesterol (HDL-C) compared to ε3 (90, 91). Epidemiological studies show that older ε4 carriers consuming diets high in saturated fat and cholesterol exhibit the worst lipid profiles, whereas switching to a low-fat, low-cholesterol diet yields disproportionately large improvements in their blood lipids (92). In other words, the *APOE*ε4 genotype is associated with an increased sensitivity to dietary fat intake. From a nutritional planning perspective, *APOE*ε4 genotyping can guide fat intake recommendations for seniors: ε4 carriers may require more restrictive limits on saturated fat and perhaps emphasis on unsaturated fats and omega-3 s (such as the Mediterranean-style diet) (93–97) to mitigate cardiovascular risk (98). Indeed, several studies suggest that reducing dietary cholesterol and saturated fat in older adults leads to significant lipid improvements and potentially lowers both cardiovascular and cognitive risk (92, 99).

Despite the promise of this approach, evidence from large studies has been mixed. A systematic review of nutrigenetic trials report inconclusive improvements in diet or metabolic outcomes, and experts note that many purported benefits of gene-based advice remain unproven in controlled trials (100). The interplay between multiple genes, nutrients, and aging processes is complex, and prospective outcome data in diverse elderly populations are still needed. Nonetheless, the principle that genomic data can refine risk reduction strategies is gaining acceptance. By integrating genetic markers (e.g., *MTHFR*, *FTO*, *APOE*, and others) with general healthy-eating principles (rich in fruits, vegetables, whole grains, lean protein, and unsaturated fats) and other lifestyle measures (physical activity, smoking cessation), dietitians and healthcare professionals can develop more individualized nutritional strategies for older adults (54).

## 4 Metabolomic profiles: real-time health snapshots

### 4.1 Metabolomics as a snapshot of nutritional health and aging

Metabolomic profiling of blood, urine or other biofluids can provide a sensitive, integrative “snapshot” of an individual’s current physiological and nutritional status (56, 101, 102). Unlike traditional diet questionnaires, metabolomic measurements capture objective biochemical features of nutrient intake and endogenous metabolism. For example, profiling of plasma or urinary metabolites can identify markers of protein intake (such as creatine or urea) and of fruit/vegetable consumption (such as proline betaine and flavonoid metabolites), allowing more accurate assessment of diet composition (103, 104). Brennan et al. emphasize that food-intake biomarkers can help mitigate measurement error in self-reported dietary intake data and enable evaluation of how diet alters metabolic pathways (105). In practice, metabolomics can reveal micronutrient deficiencies or excesses (for example low levels of B-vitamins or shifts in amino acid profiles reflecting protein malnutrition), as well as signatures of dietary pattern quality (56). These molecular indicators often predict disease risk: for instance, metabolite panels have been used to identify insulin resistance or dyslipidemia risk from elevated branched-chain and aromatic amino acids and altered lipid metabolites. Indeed, large-scale studies show that complex metabolomic signatures can stratify individuals by risk of age-related diseases and mortality better than traditional clinical factors (106–108). In effect, a person’s metabolome captures the cumulative impact of diet, lifestyle, microbiome and genetics on metabolic health, thereby signaling nutritional imbalances or early disease processes before overt symptoms appear, contributing to healthy aging.

### 4.2 Metabolomic biomarkers of aging

With advancing age, characteristic shifts in amino acid and lipid metabolites emerge in the circulation. Aging is accompanied by systemic metabolic changes, including declines in energy and redox homeostasis, that are reflected in the metabolome (109–111). Across multiple cohorts, researchers have found that elderly adults tend to have altered profiles of several amino acids: for example, circulating glycine levels often decrease with age while branched-chain amino acids (BCAAs) may rise or become dysregulated as muscle mass and insulin sensitivity decline (112–114).

Parallel lipidomic shifts have been documented as hallmarks of biological aging. Aging often associates with a decline in certain polyunsaturated fatty acids (PUFAs) and complex phospholipids, along with changes in lipoprotein composition. The study by Ali et al. (2022) investigated age-associated changes in circulating fatty acids across adults (18–64 years), older adults (65–89 years), and long-lived individuals aged 90–111 years in Southern Italy. They found that total PUFAs and trans-fatty acids declined with advancing age, while saturated fatty acid levels remained largely unchanged. Contrastingly, long-lived individuals exhibited significantly higher levels of monounsaturated fatty acids (MUFAs) compared to both adult and older adult groups (115).

These findings suggest that although aging is generally associated with decreased PUFA levels, affecting essential omega-6 and omega-3 fatty acids, senescent individuals achieving exceptional longevity often maintain elevated MUFA proportions. The authors hypothesize that this MUFA-rich fatty acid profile may help maintain membrane fluidity while reducing lipid peroxidation and oxidative damage, potentially contributing to healthier aging. Additionally, genetic variants in *FADS1/FADS2* (rs174537) and *ELOVL2* (rs953413), which regulate PUFA biosynthesis, were found to influence levels of linoleic acid and docosatetraenoic acid, indicating a genetic contribution to individual fatty acid profiles and possibly longevity (115).

These lipid changes are coupled with altered circulating lipoprotein measures (larger LDL particle size and different triglyceride profiles have been observed in children of long-lived individuals), suggesting that age-associated lipid remodeling may influence cardiovascular aging (116). Importantly, a metabolomic “age clock” built from dozens of metabolites, including amino acids, lipids, and energy intermediates, can predict mortality and frailty beyond chronological age (117, 118).

The study by Shang et al., examined “metabolomic age,” a biomarker derived from blood metabolite profiles, as a predictor of risk across 50 chronic diseases in a large UK Biobank cohort (*n* ≈ 110,692) followed for about 11–12 years (119). They trained and validated a ML model using 168 serum metabolites detected using nuclear magnetic resonance spectroscopy to calculate each participant’s metabolomic age and computed a “chronological age-adjusted age gap (CAAG)” by comparing metabolomic to actual age. A higher CAAG, indicative of accelerated biological aging, correlated strongly with increased incidence of 15 chronic conditions after multivariate adjustment, including cardiometabolic diseases (e.g., diabetes, CVD), several cancers, chronic kidney and lung disease, liver disease, and age-related macular degeneration. Notably, risk associations were stronger among those with unhealthy diets, lower education, or metabolic disorders. The findings underscore metabolomic age as a robust biomarker predictive of broad chronic disease risk, with potential for early identification and prevention strategies tailored to metabolic and lifestyle profiles. According to the mentioned study, the main metabolomic biomarkers associated with aging included various amino acids, lipids (especially very-low-density and low-density lipoprotein subclasses), glycolysis-related metabolites, ketone bodies, and inflammation-linked compounds. Elevated branched-chain amino acids (BCAAs), reduced high-density lipoprotein lipids, increased glycoprotein acetyls, and changes in lactate and citrate levels were among the most strongly predictive features of metabolomic age and its divergence from chronological age (119). Table 1 summarizes the key metabolomic biomarkers of aging.

**Table 1:** Key metabolomic biomarkers of aging and their dietary implications.

Biomarker/metabolite	Age-related change	Physiological implication	Potential dietary intervention
Glycine	Lower levels	Reduced anti-inflammatory and cytoprotective functions	Glycine-rich foods or supplements (112–114)
BCAAs	Dysregulated levels	Associated with insulin resistance and sarcopenia	Adjust protein type/amount; favor leucine-rich sources (119)
Citrulline	Lower levels	Marker of intestinal function and NO production	Prebiotics and protein balance (182)
Malate / Citrate	Altered TCA cycle intermediates	Shifts in energy metabolism and mitochondrial function	Whole grains and fruit (183)
LPCs	Lower levels	Impaired membrane integrity and inflammation control	Omega-3 fatty acid intake (184)
TMAO	Higher levels, especially with red meat intake	Linked to CVD risk	Limit red meat; increase fiber (121–123)

BCAAs, branched-chain amino acids; CVD, cardiovascular disease; LPCs, lysophosphatidylcholines; NO, nitric oxide; TCA, tricarboxylic acid cycle; TMAO, trimethylamine N-oxide.

This table summarizes key metabolites that change with age, their physiological relevance, and how their profiles can inform dietary interventions in older adults. It illustrates how metabolomics offers actionable insights for tailoring protein, fat, and micronutrient intake in precision nutrition for aging.

### 4.3 Integrating metabolomics to tailor dietary interventions

The dynamic nature of metabolomic data makes it particularly valuable for informing personalized nutrition strategies, especially in older adults, whose nutritional requirements often vary considerably due to age-related physiological changes (102). By interpreting an individual’s metabolomic profile, nutritionists can adjust diet composition, for example by modifying protein intake, to correct metabolic imbalances. Brennan et al. suggested that combining metabolite biomarkers with traditional dietary data will facilitate “effective precision nutrition” (105). For older adults, tailoring protein consumption is a prime example: metabolomics can indicate when protein intake is suboptimal by showing altered amino acid turnover markers or low levels of essential amino acids (13). In a controlled trial of elderly men, doubling dietary protein intake to approximately 1.6 g/kg/day compared with the protein RDA led to only modest shifts in the plasma metabolome. The main changes involved metabolites related to amino acid and nitrogen metabolism: urea, creatine, and glutarylcarnitine increased in the high-protein diet group, whereas the lower-protein group showed higher relative levels of glutamine and proline (120). This suggests that standard diets may already meet many metabolic needs, and that metabolomics can pinpoint which amino-acid pathways become saturated or deficient. Such findings imply that older individuals with low muscle mass or sarcopenia might benefit from targeted increases in leucine-rich proteins, whereas those with renal insufficiency might require moderation.

Metabolomics also reveals broader diet–metabolism relationships that can guide interventions. For example, shifts in microbial metabolites like trimethylamine-N-oxide (TMAO) have been linked to cardiovascular risk in older cohorts (with higher TMAO correlating with greater mortality), implying that reducing red meat intake could beneficially alter the metabolome. In practice, clinicians could measure key lipid and amino-acid metabolites to assess cardiovascular or glycemic risk and then advise dietary changes accordingly (121–123). In this context, multi-omic approaches are increasingly being developed to integrate metabolomic, genomic and microbiome data, enabling the identification of distinct metabotypes or subgroups of individuals characterized by differential responses to dietary components. Recent reviews note that metabolomic profiling is particularly useful for capturing both exogenous food-derived compounds and endogenous pathway status. By defining an individual’s metabolic phenotype (for instance, a profile of one-carbon metabolites that predicts salt-sensitivity of blood pressure), diet recommendations can be customized (56, 124–126). In older adults, where nutrient absorption and metabolism can vary widely and may be impaired, such personalization is crucial. The integration of metabolite profiles with other biomarkers, including age, kidney function, and genetic variants may facilitate the development of predictive models capable of identifying individuals who are likely to benefit from increased dietary protein intake or targeted micronutrient supplementation, as well as those who may require dietary restrictions (127).

## 5 The microbiome: a key player in healthy aging

### 5.1 Gut microbiome and healthy aging

As humans age, their gut microbial ecosystem often undergoes profound restructuring. Studies report that older adults exhibit lower microbial diversity and evenness than younger individuals (128–130). Typical patterns include loss of beneficial commensals (such as *Bacteroides* and *Bifidobacterium*) and relative expansion of facultative or opportunistic taxa (for example, members of *Proteobacteria* like *Escherichia coli*) (131–133). These compositional shifts reflect interacting host and environmental factors. Many elderly individuals consume less plant-derived fiber and fewer phytonutrients, due to changes in appetite, dentition or gastrointestinal function. In parallel, increased use of medications (antibiotics, proton pump inhibitors) and reduced physical activity in older age alter gut conditions (134, 135).

### 5.2 Dietary impacts on the aging microbiome

Nutrition remains a dominant driver of gut ecology in older adults (136). Diets high in fermentable plant fibers and phytonutrients tend to preserve a diverse, resilient microbiome, whereas age-related shifts toward processed, high-fat and high-sugar foods accelerate imbalance (137–140). For example, greater adherence to Mediterranean or other plant-rich dietary patterns is associated with higher microbiome diversity and a greater abundance of beneficial genera (e.g., *Bacteroides*, *Faecalibacterium*) in older populations (140–142). Conversely, habitual low intake of non-digestible carbohydrates correlates with declines in SCFA producers (143). Dietary (poly)phenols also play a modulatory role: plant polyphenols, found in berries, tea, spices, and other plant foods, reach the colon largely intact, where microbial enzymes transform them into bioactive metabolites. These compounds may suppress pathogenic microorganisms and create ecological conditions that favor symbiotic taxa, including *Akkermansia* and butyrate-producing bacteria (144). Plant-derived bioactive compounds may also contribute to intestinal barrier integrity (145). However, this effect should not be interpreted as uniformly beneficial under all dietary conditions. *Akkermansia muciniphila* is a mucin-degrading species, and when dietary fiber is insufficient, increased reliance on host mucin as a substrate may contribute to mucus layer thinning and increased intestinal permeability, or ‘leaky gut’. Therefore, barrier protection in older adults likely depends on a balanced microbial network that includes SCFA-producing, particularly butyrate-producing, genera such as *Roseburia* and *Blautia*, adequate fermentable fiber intake, and, where appropriate, probiotic or synbiotic strategies that support epithelial integrity rather than the expansion of a single taxon in isolation (146).

### 5.3 Biotic-based strategies for healthy aging

**Figure 1:**
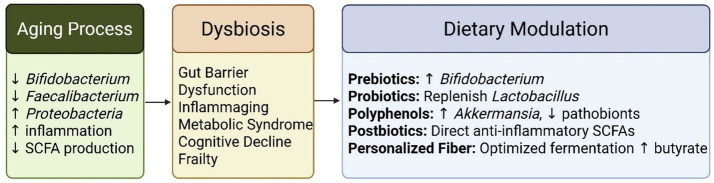
Age-associated gut microbiome shifts and dietary modulation pathways.

Several microbiome-targeted intervention strategies are being explored to promote a youthful gut profile in elders (147). Probiotics (supplements of live beneficial microbes) are among the most studied: trials in older adults report that supplementation with *Lactobacillus* and *Bifidobacterium* strains can partially restore beneficial taxa, improve gut barrier function, and reduce circulating inflammatory markers (148, 149). Similarly, prebiotic interventions (administration of non-digestible fibers such as inulin, fructooligosaccharides or resistant starch) selectively feed commensal bacteria (150, 151). In elderly cohorts, prebiotics have been shown to increase *Bifidobacterium* and butyrate-producing bacteria and to improve metabolic health indices. Synbiotic products combining probiotics and prebiotics often yield additive benefits. Beyond conventional formulations, next-generation prebiotics and probiotics are being investigated as more targeted microbiome-modulating strategies. These approaches aim to restore specific microbial functions relevant to healthy aging, including SCFA production, immune regulation, metabolic homeostasis, and intestinal barrier support. However, their application in older adults requires careful consideration of strain-specific effects, baseline microbiome composition, frailty, comorbidities, medication use, and safety (48). In parallel, postbiotics and paraprobiotics (microbial metabolites or inactivated microbes) have emerged as interventions that may confer some probiotic benefits with greater safety (Figure 1) (152, 153).

This conceptual figure illustrates how the gut microbiome changes across the aging process—characterized by reduced microbial diversity, loss of beneficial taxa, and increased inflammatory species—and how specific dietary strategies (fiber, prebiotics, polyphenols, probiotics, postbiotics) can modulate these changes to promote healthy aging. The flow visualizes the interaction between age, gut dysbiosis, health outcomes (e.g., inflammation, frailty), and dietary interventions that restore microbial balance (131–133). Abbreviations and symbols: SCFAs, short-chain fatty acids; ↑ indicates increase; ↓ indicates decrease.

### 5.4 Personalized fiber and polyphenol recommendations

Beyond general interventions, there is a growing emphasis on tailoring dietary strategies to an individual’s unique gut ecology (152). Cutting-edge metagenomic analyses can now infer an individual’s repertoire of fiber-degrading enzymes, enabling personalized fiber prescriptions. For instance, if a person’s microbiome lacks enzymes to break down certain polysaccharides, alternative fermentable fibers can be recommended to optimize SCFAs production and beneficial microbial growth (154–156). A similar precision approach applies to polyphenols: gut bacteria differ in their ability to metabolize plant compounds, so that (poly)phenol-rich foods may need to be matched to one’s microbial profile. Notably, only specific commensals harbor enzymatic pathways (such as tannases or phenolic acid reductases) to convert dietary phenolics into bioactive metabolites (157–159). (Poly)phenols thus function as “duplibiotics,” exerting both antimicrobial effects on opportunists and prebiotic-like stimulation of symbionts (158). The implication is that the real exposure to dietary (poly)phenol intake is influenced by many factors like the person’s age, genetics, gut microbiome and health status.

## 6 Personalized nutrition models in clinical practice

As the global population ages, healthcare systems are increasingly investing in personalized nutrition models as part of broader strategies to prevent chronic disease, reduce healthcare costs, and maintain functional independence in older adults (160). Precision nutrition, defined as the use of individual biological and lifestyle data to inform tailored dietary recommendations, has moved from theoretical frameworks into early clinical implementation (39, 161). These models often integrate data from genomics, metabolomics, microbiome profiling, and digital health tracking, producing individualized dietary plans aimed at optimizing metabolic health and reducing age-related disease risk (41, 162–164). Several pioneering clinical programs and trials have begun validating these approaches in real-world settings, particularly among aging populations at risk of metabolic syndrome, cognitive decline, and frailty (164).

One of the most prominent examples of a large-scale precision nutrition initiative is the PREDICT (Personalized Responses to Dietary Composition Trial) study, a multi-phase program led by researchers at King’s College London and the Harvard T.H. Chan School of Public Health (165). PREDICT2, the second phase of this trial, involved more than 1,000 individuals and used deep phenotyping, including postprandial metabolic responses, gut microbiome composition, and genetic variants, to predict personalized dietary responses (166). The findings of PREDICT demonstrated that individuals exhibit wide variability in their glycemic, lipid, and inflammatory responses to the same foods, largely independent of calorie content. Importantly, ML models developed from these data successfully predicted individual postprandial responses based on multi-omic features (165). The insights from PREDICT are now being translated into digital nutrition tools (e.g., ZOE app), which offer real-time personalized dietary advice and metabolic feedback, potentially making the science accessible to consumers and clinicians alike (167).

Another significant project, Food4Me, a European Union–funded randomized controlled trial, explored whether different levels of personalized nutrition advice (from general guidelines to gene-based recommendations) affected dietary behavior over 6 months. While not elderly-specific, subgroup analyses demonstrated that personalized interventions, especially those involving phenotypic feedback, produced significantly greater improvements in dietary quality and cardiovascular risk factors than conventional advice (71, 127).

In older populations specifically, clinical trials targeting cardiometabolic and cognitive decline have begun to incorporate precision nutrition frameworks (168). In this line, the NU-AGE project, which included over 1,200 older adults across five European countries, applied personalized dietary counseling based on nutritional biomarkers and regional food patterns. After 1 year, participants showed improved adherence to nutrient targets and reduced markers of low-grade inflammation, key contributors to frailty and age-related disease (169). The integration of digital technologies has further accelerated the application of precision nutrition in clinical and community settings (161). Wearable glucose monitors, digital food diaries, and AI-powered decision support systems now allow practitioners to tailor interventions in near-real time (170–172).

Together, these clinical models illustrate the translational potential of personalized nutrition, particularly its capacity to integrate biological, behavioral, and digital data into more individualized strategies for preserving metabolic health and functional capacity in aging populations.

## 7 Ethical, social, and practical challenges

As personalized nutrition becomes increasingly integrated into clinical practice, several ethical, social, and practical challenges must be addressed to ensure its effective and equitable implementation (65). Although precision nutrition offers the potential to provide individualized dietary recommendations based on genomic, metabolic, and microbiome data, barriers such as high cost, limited insurance coverage, and technological requirements continue to restrict accessibility (173), particularly among older adults with fixed incomes, limited healthcare resources, or reduced access to specialized diagnostic testing (42). Limited digital literacy, unequal access to health technologies (174), cognitive limitations, insufficient caregiver support, and insufficient clinician training in genomics and bioinformatics may further hinder the adoption of omics-informed nutritional approaches (175, 176). In addition, the collection and use of large volumes of personal health data raise important concerns regarding privacy, informed consent, data security, secondary data use, data governance, and the potential discriminatory misuse of sensitive omics information (177).

Equity remains a central consideration in the development of personalized nutrition. Most existing models have been derived from data sets composed predominantly of individuals of European ancestry (39), which limits their applicability across diverse ethnic, cultural, and geographical populations (161). Expanding representation in omics research is therefore essential to improve the generalizability and clinical relevance of personalized nutrition strategies (178, 179). Equally important is the need to design digital and omics-informed interventions that are accessible, understandable, and culturally appropriate for older adults across different socioeconomic and care contexts (180). At the same time, emerging technologies, including wearable sensors and continuous monitoring systems, may offer new opportunities for real-time assessment of dietary responses and health outcomes (181). Ultimately, the successful implementation of personalized nutrition will depend on balancing technological innovation with robust ethical oversight, inclusive research practices, and a sustained commitment to addressing the diverse needs of aging populations (180).

## 8 Conclusion

Personalized nutrition represents a significant advancement in nutritional science, offering the potential to tailor dietary recommendations based on individual genetic, metabolic, and microbiome profiles. This approach aims to optimize health outcomes and manage chronic diseases more effectively by considering the unique biological features of each individual.

Despite its considerable potential, personalized nutrition faces several challenges that must be overcome to enable its effective implementation and maximize its benefits. Accessibility remains a significant limitation, as the technologies and tests required can be costly and may not be readily available to all populations. Scalability is another issue, as implementation on a large scale requires substantial resources and infrastructure. Furthermore, more research is needed to understand the long-term health outcomes associated with personalized dietary interventions, particularly in diverse populations.

Finally, while personalized nutrition holds transformative potential for promoting healthy aging and managing chronic diseases, efforts must be made to overcome barriers related to accessibility and scalability, and to strengthen evidence of long-term benefits. Continued interdisciplinary research and collaboration are essential to develop effective, equitable, and sustainable personalized nutrition strategies.
